# Prediction of KIR3DL1 and human leukocyte antigen binding

**DOI:** 10.1016/j.jbc.2025.110437

**Published:** 2025-07-01

**Authors:** Martin Maiers, Yoram Louzoun, Philip Pymm, Julian P. Vivian, Jamie Rossjohn, Andrew G. Brooks, Philippa M. Saunders

**Affiliations:** 1 CIBMTR® (Center for International Blood and Marrow Transplant Research), NMDP, Minneapolis, Minnesota, USA; 2 Department of Mathematics, Bar-Ilan University, Ramat Gan, Israel; 3 Infection and Immunity Program and Department of Biochemistry and Molecular Biology, Biomedicine Discovery Institute, Monash University, Clayton, Victoria, Australia; 4 Institute of Infection and Immunity, Cardiff University, School of Medicine, Cardiff, UK; 5 Department of Microbiology and Immunology, University of Melbourne, The Peter Doherty Institute for Infection and Immunity, Parkville, Australia

**Keywords:** natural killer cells, major histocompatibility complex (MHC), cell surface receptor, receptor structure-function, transplantation, bioinformatics

## Abstract

KIR3DL1 is a polymorphic inhibitory receptor on natural killer (NK) cells that recognizes HLA class I allotypes. While the Bw4 motif spanning residues 77 to 83 is central to this interaction, structural studies have shown that polymorphisms elsewhere in the HLA molecule also influence binding. To address the challenge of predicting interactions across the extensive diversity of both KIR3DL1 and HLA, we developed a machine learning model trained on binding data from nine KIR3DL1 tetramers tested against a panel of HLA class I allotypes. Multiple models were evaluated using different subsets of HLA sequence features, including the full α1/α2 domains, the Bw4 motif, and α-helical residues excluding loop regions. The best-performing model, using Multi Label Vector Optimization (MLVO) and trained on α-helix positions, achieved AUC scores ranging from 0.74 to 0.974 across all KIR3DL1 allotypes. The model effectively distinguished high and low binders, revealing that residues beyond the Bw4 motif contribute to binding strength in a nonadditive manner. These findings demonstrate that binding affinity cannot be accurately captured by binary classifiers or single-motif rules. Our approach offers a more nuanced framework for modeling KIR3DL1-HLA interactions, with broad applicability to immunogenetic research and clinical decision-making.

Host genetic variation plays a critical role in shaping immune responses and disease susceptibility. Among the most polymorphic and clinically relevant gene families are the Human Leukocyte Antigens (HLA), which present peptides to the immune system (1). For example, HLA-B∗57:01 offers protection against HIV but also predisposes individuals to abacavir hypersensitivity (2, 3). Over 27,000 HLA class I alleles have been identified, encoding more than 15,000 distinct allotypes (4). While this diversity enhances adaptive immune responses at the population level (5), it poses a challenge to the innate immune system, which also relies on HLA for immune surveillance.

Natural killer (NK) cells, key effectors of innate immunity (6), recognize subsets of HLA class I molecules *via* the Killer-cell Immunoglobulin-like Receptor (KIR) family. KIR genes exhibit considerable diversity both in gene content and in allelic variation; individuals may carry between 7 and 14 activating or inhibitory KIR genes (7, 8), and more than 2200 alleles have been reported (4). Both HLA and KIR allelic variation can influence the strength of their interaction, which in turn modulates NK cell activation (9, 10). However, predicting the affinity of these highly polymorphic receptor-ligand interactions remains difficult.

This complexity is exemplified by the inhibitory receptor KIR3DL1, which binds to HLA class I molecules carrying the Bw4 motif (11) (residues 77–83 on the α1 helix). Early studies suggested that HLA-Bw4 allotypes with isoleucine at position 80 (Bw4-80I) bind KIR3DL1 more strongly than those with threonine (12) (Bw4-80T). Yet exceptions such as HLA-A∗25:01, a Bw4-80I allotype with weak KIR3DL1 binding, revealed limitations of this binary classification. Structural studies of KIR3DL1 in complex with HLA-B∗57:01 demonstrated that residues outside the Bw4 motif—and even the HLA-bound peptide—also contribute to binding, highlighting a broader structural basis for interaction (13, 14, 15, 16, 17, 18, 19).

KIR3DL1 itself is highly polymorphic, with over 300 alleles encoding 138 distinct protein variants. These span three main phylogenetic lineages, including two inhibitory lineages comprising *KIR3DL1∗005*-like and *∗015*-like alleles, and a third lineage which is much more constrained at a population level and consists primarily of the activating *KIR3DS1∗013* allele (20).

Allotypes differ in cell surface expression and HLA binding capacity; for instance, KIR3DL1∗005 binds a broader set of HLA allotypes than KIR3DL1∗015 (9, 16), and some allotypes like 004 are poorly expressed on the cell surface (10, 21). Differences in key residues, such as positions 238 and 283, have been associated with functional divergence in HLA recognition (15, 22, 23, 24). These molecular features contribute to distinct binding hierarchies, where some HLA-Bw4 allotypes, like B ∗ 57:01 are recognized broadly, while others, such as A ∗ 24:02, are selectively bound by specific KIR3DL1 variants.

These interactions are functionally important. KIR-HLA combinations influence NK cell education, a process that enables discrimination between healthy and diseased cells, especially in contexts like infection, cancer, and allogeneic transplantation. Consequently, genetic studies have examined KIR/HLA pairings in clinical outcomes, including treatment responses in leukemia (25, 26, 27, 28) and neuroblastoma (27, 28) as well as disease progression in HIV (29). These studies typically use simplified metrics—Bw4-80I *versus* 80T, or KIR3DL1 expression level—to classify interactions as strong or weak. However, both experimental and clinical data increasingly reveal exceptions to these categorizations. Binding strength exists on a spectrum, and many interactions fall outside binary "binder/non-binder" thresholds.

Compounding this complexity is the lack of experimental binding data for many KIR3DL1-HLA combinations (30). The polymorphism of both genes and the uneven representation of HLA allotypes across populations make it impractical to test all possible interactions *in vitro*. To address these limitations, we developed a machine learning model to predict the strength of KIR3DL1 binding to HLA class I allotypes using amino acid sequence features. This continuous, data-driven model enables broader and more precise application of KIR-HLA interactions in immunogenetic research and clinical outcome prediction.

## Results

### Poor population coverage of empirical KIR/HLA binding data

**Figure 1:**
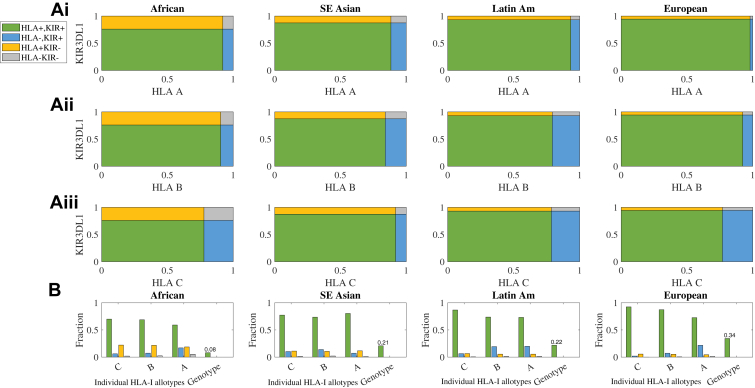
**Available KIR3DL1/HLA-Bw4 binding data disproportionately covers populations.***A*, the frequency of HLA-A (i), HLA-B (ii) and HLA-C (iii) allotypes, along with KIR3DL1 allotypes covered by the present KIR3DL1/HLA-Bw4 binding data among African, Asian, Latin American and European populations *Green* regions represent the fraction of the population where both HLA-I and KIR3DL1 binding information are covered, *yellow* represents only KIR3DL1 coverage and *blue* only HLA-I coverage, while *gray* is where neither are present in the current datasets. *B*, summary of the covered HLA-I allotypes along with the fraction of the HLA-I genotypes (two of each HLA-A, HLA-B and HLA-C) fully covered by the present experimental binding measures. For all populations, assuming independence, this fraction is below 0.35.

To first demonstrate the requirement for a KIR/HLA binding prediction tool, the fraction of the population covered by the current experimental panel of KIR3DL1 and HLA-I was evaluated (Fig. 1). At present, the binding of three tetrameric KIR3DL1 allotypes (∗001, ∗005, and ∗015) and seven KIR3DL1-tetramers or -Fc allotypes (∗005, ∗007, ∗001, ∗002, ∗015, and ∗020) to HLA-coated beads has been examined (9, 31). This panel is here extended, with the additional binding of nine tetrameric KIR3DL1 allotypes (∗001, ∗002, ∗004, ∗005, ∗008, ∗009, ∗015, ∗020, and ∗029) measured analogously. Despite this broad panel, based on allele frequencies in the literature, 6 to 24% of known KIR3DL1 alleles (32, 33, 34) and 11 to 23% of HLA-I alleles (35) remain untested. On average, 76% of the KIR3DL1/HLA-I allele combinations were covered (Fig. 1*A*, green rectangles). This coverage was maximal in European and Asian populations and was less in African populations, as expected due to their greater genetic diversity (Fig. 1*A*).

The implications of this coverage challenge for KIR3DL1/HLA-I allotypic combinations are worse when considering full HLA class I genotypes. Since each individual expresses two HLA-A, -B, and -C alleles, the proportion fully covered in the experimental panel reduces to 8% in African populations, and to 34% in European populations (Fig. 1*B*). Thus, even in the best-covered population, two-thirds of individuals have at least one HLA allele for which experimental data are lacking. To address this gap, we developed two types of predictive models: a prediction tool for a KIR3DL1 allotype, which has been empirically tested (the nine alleles) but not against the HLA in question, and a second for when there are no experimental data for the KIR3DL1 allotype.

### HLA-I binding to KIR3DL1 forms three clusters

Inhibitory KIRs share a common binding site on HLA-I spanning residues in both the *α*1 and *α*2 helices. Notably, KIR2DL1/2/3 and KIR3DL1 all bind to residues 145, 146, and 149 to 151, all of which are highly conserved across HLA-A, -B, and -C allotypes (36, 37). The other major KIR docking site spans residues 69 to 84, which incorporates both the C1 and C2 serological determinants as well as the determinants of the Bw4 and Bw6 epitopes (38). Position 80 along with residue 77, are considered the specificity-determining residues for KIR2DL1 *versus* KIR2DL2/3 recognition of HLA-C2 and C1 molecules, respectively (39, 40), while positions 80 and 83 are important in KIR3DL1 recognition of HLA-Bw4 molecules (16, 41). Among the HLA-I bead binding panels, there were five combinations of residues 80 and 83, two of which corresponded to Bw4 allotypes (I80/R83 and T80/R83), K80/G83 and N80/G83 denoted C1 and C2 allotypes respectively while T80/G83 characterized the bulk of HLA-A allotypes (Table 1). Notably, HLA-B molecules with the Bw6 motif also exhibit the N80/G83 combination seen in C2 allotypes. This supports the structural similarity between these subgroups.

**Table 1:** Class I common polymorphisms at positions 80 and 83 by epitope name and locus

Pos80	Pos83	C1/C2/Bw4	Loci
N	G	C1	B C
T	G	-	A
I	R	Bw4	A B
T	R	Bw4	B
K	G	C2	C

**Figure 2:**
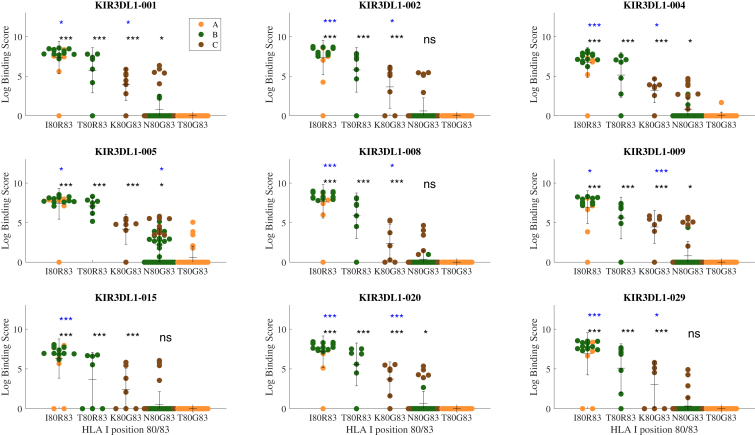
**HLA-I residues 80 and 83 impact KIR3DL1 binding patterns.** The log binding score plotted for each tested HLA-I allotype (*yellow* HLA-A, *green* HLA-B and *red* HLA-C) for nine KIR3DL1 allotypes. HLA-I are grouped by their amino acid residue combinations at positions 80 and 83 (very rare combinations were ignored). Each group is compared to the lowest binding group, T80/G83 (in *black* ∗ *p* < 0.05, ∗∗*p* < 0.01,∗∗∗*p* < 0.001,ns Non-Significant). Additionally, the *blue* stars represent the same *p* value ranges but comparing each residue combination to the one to its *right* and using a Tukey test.

To first broadly compare how the combinations of residues 80 and 83 impact HLA/KIR binding, the binding of nine distinct KIR3DL1 allotypes to these 97 HLA-I allotypes was assessed. As expected, regardless of KIR3DL1 allotype, HLA-I allotypes that possessed either I80/R83 or T80/R83 exhibited superior binding to KIR3DL1 tetramers compared to other 80/83 combinations (Fig. 2). Interestingly, the K80/G83 (C1) and HLA-C encoded N80/G83 (C2) combinations, while on average not binding as well as the Bw4 allotypes, nevertheless showed significant reactivity with KIR3DL1 compared with the T80/G83 allotypes (Mann–Whitney test between K80/G83 or N80/G83 and T80/G83 *p* < 0.001 for all KIR3DL1 allotypes). Additionally, there were significant differences in recognition patterns across individual KIR3DL1 allotypes, with KIR3DL1∗005 being the most distinct, as evidenced by a greater capacity to bind N80/G83 allotypes and even several T80/G83 allotypes. Nevertheless, HLA allotypes with I80 and T80 (both with R83) were associated with high binding to KIR3DL1. K80 and N80 can be either low-level binders (typically on HLA-C allotypes, but some HLA-B allotypes, mainly to KIR3DL1∗005) or non-binders (typically on HLA-B allotypes), and T80/G83 do not bind to KIR3DL1, except for KIR3DL1∗005. The variation in KIR3DL1 binding within each of these HLA-I subgroups therefore reinforces the notion that polymorphisms outside of regions 77 to 83 of the *α*1 helix can significantly impact HLA class I recognition by KIR3DL1.

**Figure 3:**
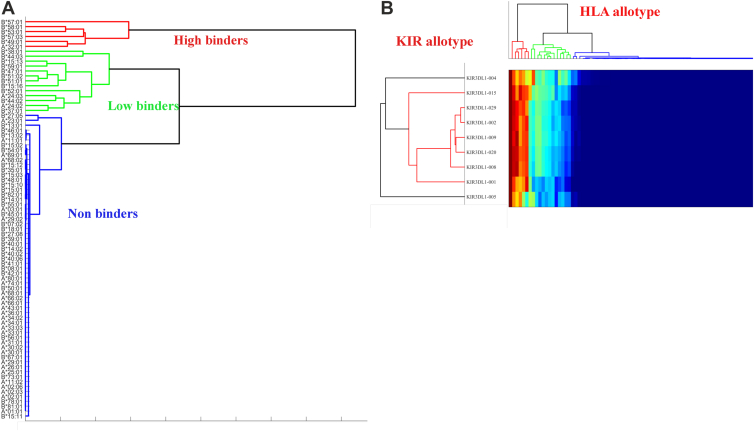
**KIR3DL1∗005 and ∗004 are more promiscuous in binding HLA-I.***A*, clustering of the HLA-I allotypes in terms of their KIR binding behavior. *B*, two-dimensional clustering of HLA-I allotypes across all nine KIR allotypes revealing *high* binders (*red*), *low* binders (*green*) and non-binders (*blue*).

To compare the diversity of binding patterns, we performed hierarchical clustering on KIR allotypes in terms of their binding to HLA allotypes. KIR3DL1 allotypes ∗002, ∗008, ∗009, ∗020, and ∗029 were highly similar in their recognition of HLA-I, while KIR3DL1∗005 and ∗004 were the most divergent (Fig. 3). These differences only partially matched the sequence phylogeny with KIR3DL1∗002, ∗008, ∗009, ∗020, and ∗029 in the ∗015-like lineage and ∗004 and ∗005 in the ∗005-like lineage (33). Curiously, KIR3DL1∗015 binding to HLA-I sat somewhat apart from the rest of its lineage representatives.

To better define the residues in HLA-A and -B that were responsible for differences in KIR3DL1 binding clustering of HLA-I allotypes was performed considering their binding by all KIR3DL1 allotypes (*i.e.* each HLA-I allotype is represented by its vector of log binding affinities to each KIR3DL1 allotype, normalized for each KIR allele between 0 and 100). Three clusters of binding were observed based on the top three clusters of average link hierarchical clustering representing high binders (such as HLA-B∗57:01, -B∗58:01 and -A∗32:01), low binders (including HLA-B∗38:01, -B∗51:01, -B∗44:02 and -A∗24:02), and non-binders (mostly comprised of HLA-Bw6 and HLA-A allotypes, but also including HLA-Bw4 allotypes like HLA-B∗27:05, -B∗13:01 and -A∗25:01) (Fig. 3*A*).

**Figure 4:**
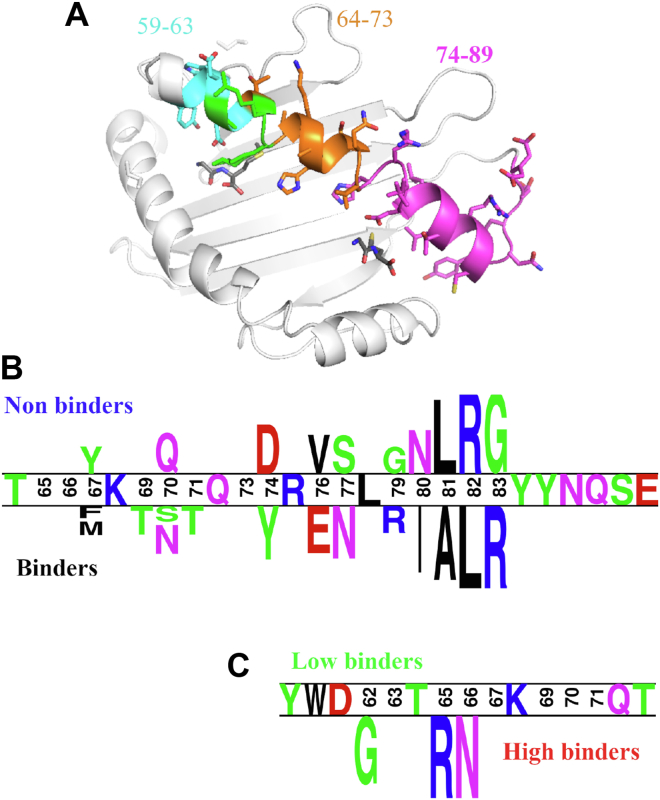
**Residues beyond the Bw4 motif impact KIR3DL1 binding.***A*, regions on the a1 domain of the HLA class I molecule (PDB accession 6TDQ) contributing to binding. The *purple* residues comprise positions 74–89 which were the most significant in the binding prediction. All colored positions are the no-loop regions. *Blue* residues are positions 59–63, which were significant for the *low* to *high* binder predictions, and *orange* residues are 64–73 that were important for both predictors. Position 62, 65 and 66 that affect *low* vs *high* binding are indicated in *green*. Positions not colored showed no significant enrichment or depletion. *B*, logo diagram of the amino acid residues in HLA-A and HLA-B allotypes with the largest enrichment or depletion associated with binding vs non-binding (both *high* and *low*) and (*C*) *high*- *versus low*-binding allotypes. The size of each letter is proportional to its impact.

All HLA-I allotypes classified as high or low binders (red and green clusters) were next compared to the non-binding cluster (blue cluster), and the amino acid residues significantly contributing to the binding were computed. When split using a two-population logo plot, where the size of each letter is proportional to its contribution to the difference between the populations (Fig. 4*B*), the contribution to binding was highest at positions 80 to 83, with a clear IALR (Bw4) signature motif for binders and NLRG (Bw6) motif for non-binders. However, there were also significant contributions from positions extending back to residue 67, which may be due to linkage disequilibrium. Interestingly, not all HLA allotypes that bind KIR3DL1 have the full IALR signature (such as HLA-B∗44:02, which has a Thr at position 80), but each of these residues contributes significantly to the difference between binders and non-binders. To further detect the residues associated with the distinction between low and high binders, we performed a similar analysis focusing only on the differences between the high and low binders (Fig. 4*C*). The amino acid residues that significantly contributed to this distinction were at positions 62, 65, and 66 (highlighted in green on Fig. 4*A*), with four of the six high-binding HLA-Bw4 allotypes possessing Arg65 and Asn66. Notably, these residues all form part of the A pocket that accommodates peptides.

The most significant regions for KIR3DL1 binding were the outward-facing amino acids in the *α*1 helix, particularly the classical Bw4 motif located at the C-terminal end of the *α*1 helix (Fig. 4*A*). Regions further from the C-terminal end have a lower, yet significant (at the *p* = 0.05 level), influence on KIR3DL1 binding. Importantly, all amino acids within the *α*1 and *α*2 domains were considered in the analysis. However, only residues in the *α*1 helix had variation substantial enough to produce a signal. This does not preclude the impact of unique polymorphisms on the *α*2 helix on specific allotypes such as positions 145 for HLA-B∗13:01 and 149 for HLA-A∗25:01 that have been reported to impact KIR3DL1 recognition (18). Nevertheless, these clustering analyses identified three groups of HLA-I binding to KIR3DL1, high, low, and non-binders, with their binding strength influenced by residues outside of the Bw4 motif on the *α*1 helix.

### A tool to predict KIR3DL/HLA-I binding

**Figure 5:**
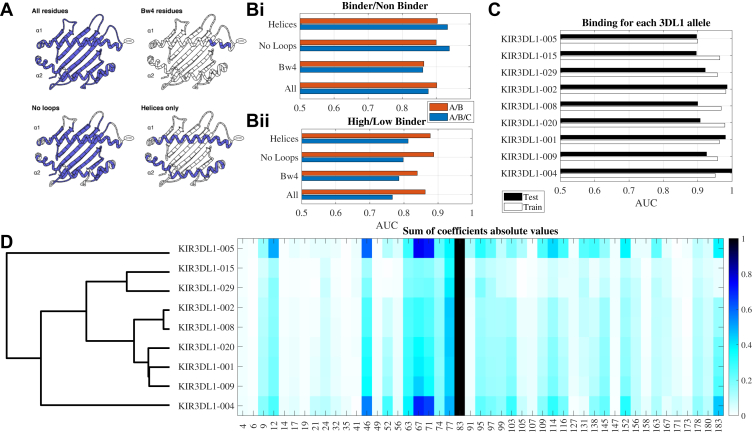
**Modeling with the HLA-I helices gives the greatest accuracy.***A*, representation of the different sets of positions in the HLA-I molecule tested. *B*, test AUC of classification into binders and non-binders (*i*) or *high* and *low* binders (*ii*) for each tested HLA-I region and using either all loci (*A*/*B*/*C*) or only the HLA-A and HLA-B loci (*A*/*B*). All KIR3DL1 allotypes were used simultaneously. Each HLA allotype was either in the training or test set for all allotypes. *C*, AUC of classifier per KIR3DL1 allotype using the no-loops and all HLA loci option. All AUC results in plots *B* and *C* are fivefold cross validation. *D*, sum of absolute value of coefficients normalized by min-max (maximal value is 1, minimal value is 0). Each row is a classifier, and each column is a position. Only variable positions are marked. The allotypes are clustered and ordered according to their clustering (dendrogram to the *left*).

Although the empirical measurements of KIR3DL1/HLA-I binding identify differences in their strengths of interaction, they cannot account for the vast polymorphism of both *KIR* and *HLA* at a population level. Therefore, both linear and non-linear kernel methods were used to predict the strength of interaction between the KIR3DL1 allotypes and *any* HLA class I allotype. Disease association studies attempting to link KIR3DL1/HLA-Bw4 interactions with clinical outcomes have typically only considered the presence or absence of the Bw4 motif or the presence of an Ile or Thr at position 80. Consequently to assess the broader contribution made by HLA-I residues on this interaction, the performance of each model was evaluated using four different options for the inclusion of amino acid residue positions: 1) all positions encoding the *α*1 and *α*2 domains (all), 2) all positions except connecting loops (no loops), 3) only positions on the alpha helices (helices), and 4) only the six positions that contribute to the Bw4 motif itself (Bw4) (Fig. 5*A*). Since Bw4 binding motifs are confined to HLA-A and B molecules (12), the models were also evaluated with and without the inclusion of HLA-C (A/B/C and A/B).

While dimensionality reduction for categorical variables is often performed using Multiple Correspondence Analysis (MCA), we found that Principal Component Analysis (PCA) yielded better model performance, with significantly higher AUC values (*t* test *p* < 0.05). Similarly, both Multi Label Vector Optimization (MLVO) and Support Vector Machine (SVM) classifiers produced higher AUCs, with MLVO achieving the best accuracy overall, as measured by the Area Under the Curve (AUC), where a score of 1 indicates perfect separation between binding categories (Fig. S5). More complex models, including XGBoost and neural networks, were also tested but showed lower test set AUCs. The superior performance of linear classifiers suggests that the effect of individual residues on binding strength is largely additive when modeled on the logarithmic scale.

The prediction of binders and non-binders was next tested, based on the information from all KIR3DL1 allotypes (see methods for training/test division) (Fig. 5*B*i). When comparing different types of input, there was a significant difference between the input types (two-way ANOVA *p* < 0.05). There was no effect in the comparison between the loci used (A/B vs A/B/C) for the overall binding. However, there was an effect depending on the HLA-I amino acid residues included in the model. Use of the six positions that contribute to the Bw4 motif led to a lower accuracy of the model (AUC value) than the alternatives when considering HLA-A and B, and thus reinforced the importance of residues beyond this motif in determining KIR3DL1 binding (Tukey test *p* < 0.05, Fig. 5*B*i).

A similar analysis was performed to predict the high/low-binder categories, using a linear classifier (Fig. 5*B*ii). Overall, the accuracy of the model predicting low from high binders was lower than that for the binders and non-binders. Indeed, removing HLA-C from the analysis significantly reduced the performance of the high to low binders (ANOVA *p* < 0.05, Tukey test <0.05 on all combinations of groups without HLA-C *versus* groups with HLA-C). This is expected based on the data in Figure 2, where many of the low-level binders are HLA-C allotypes. However, even when HLA-C was removed, a partial classification could still be obtained, since even within the HLA-A and -B allotypes there are differences between high and low-binders. Again, the two-way ANOVA was significant (*p* < 0.05), with both the loci (with and without C) and the input type was also significant (*p* < 0.05).

We further used only positions in the helices (including HLA-C), which was the minimal model with the highest accuracy separating binders and non-binders, to predict HLA-I binders *versus* non-binders for each KIR3DL1 allotype separately (Fig. 5*C*) with the training cases (white bars) and test cases (black bars). Interestingly, a clear difference was found between allotypes with KIR3DL1∗005 performing worse, and other KIR3DL1 allotypes like ∗002 having a test set AUC of almost 1, all based on the same number of observations (ANOVA *p* < 0.01, *p* < 0.01 Tukey test of KIR3DL1∗005 *versus* others). The divergent, broader binding pattern of KIR3DL1∗005 likely drove its suboptimal performance in the model, although KIR3DL1∗004 performed better despite also having a divergent binding pattern. The linear classifier was then used to estimate the contribution of each HLA-I amino acid position to the binding prediction (Fig. 5*D*). The total contribution of each position was normalized to 0, and the average contribution of amino acids in each position calculated. As expected, residues comprising the Bw4 motif (77–83) distinguished HLA-I binders from non-binders across all KIR3DL1 allotypes, with additional positions across the *α*1 and *α*2 helices contributing. Notably, residues 67, 71, and 49 in the HLA-I contributed mainly to KIR3DL1∗005 and ∗004 recognition of binders and non-binders and is again consistent with the different binding patterns (Fig. 3) and classifiers for these two allotypes.

### Testing the KIR3DL1/HLA-I binding predictor

**Figure 6:**
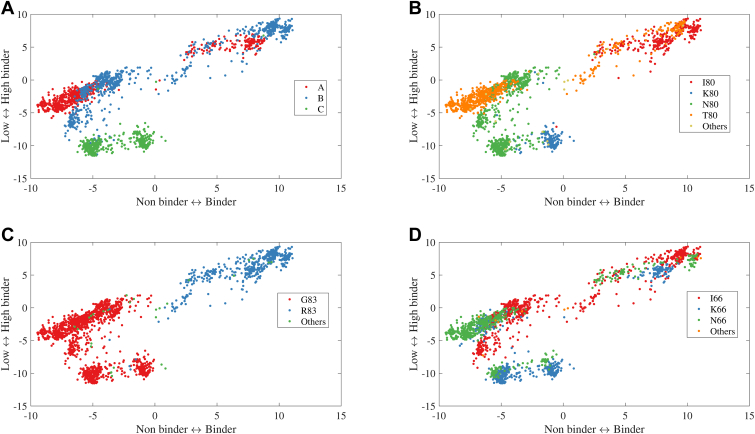
**HLA-I type, plus residues at positions 80 and 83, predict the strength of KIR3DL1 binding.** Prediction was performed for all HLA allotypes of the binder/non binder classifier (x axis) and *high*-*low* binding classification (y axis). HLA-I allotypes were distinguished by type (HLA-A, -B or -C) (*A*) or by the amino acids present at position 80 (*B*), 83 (*C*) and 66 (*D*). Rare combinations were removed.

To date, more than 6000 HLA-B proteins have been described, around a third of which carry the Bw4 epitope while about 20% of the almost 5000 HLA-A allotypes reported are Bw4^+^ (4, 41). To assess the capacity of the model to assign binder/non-binder and low/high binding classifications to additional, untested HLA-I allotypes, the binding scores to all KIR3DL1 allotypes were computed for 8000 HLA-A, -B, and -C allotypes (Fig. 6). Grouping by class I type, non-binders were mostly HLA-A allotypes, while the high binders contained both HLA-A and -B allotypes and the low binders were mostly HLA-C allotypes (Fig. 6*A*). Overall, HLA-B allotypes tended to have higher predicted binding probability than HLA-A allotypes. When looking at the amino acid composition, binding corresponded primarily with the presence of the Bw4-distinguishing residue R83 (Fig. 6*C*), as well as I80 and T80-containing motifs (Fig. 6*B*). Notably, both residues at position 80 were predictive of high binding, suggesting that segregation of weak and strong KIR3DL1 ligands on the basis of this one residue alone is imprecise. Some HLA-A allotypes with I80/R83 were non-binders (*e.g.* HLA-A∗25:01), yet other HLA-A along with HLA-B allotypes with I80/R83 were regarded as high binders. Additionally, other T80 allotypes originating from HLA-A (G83) did not bind at all while C1 (N80/G83) and C2 (K80/G83) molecules sat in the middle range. Position 66 was additionally assessed due to its variation significantly contributing to the distinction of high and low binders in the clustering; however, no single amino acid was found associated with high *versus* low binding (Fig. 6*D*). Therefore, beyond the broad distinctions between the main KIR ligands, the current analysis gives a much more calibrated representation of KIR3DL1 and HLA-I binding.

To further test the accuracy of the predictor on unseen HLA allotypes, we used previously published mutation studies examining the recognition of wild type and mutant HLA by KIR3DL1^+^ NK cells. In these studies, HLA-I molecules were mutated at specific residues and transfected into 721.221 (221) cells, which lack endogenous HLA-A and -B allotypes. Purified NK cells from healthy blood donors typed for KIR3DL1 were then incubated with HLA-expressing 221 target cells and degranulation measured after 5 hours by flow cytometry. The mutations examined included residues comparing the recognition of HLA-B∗57:01 and -B∗13:01 (residue 145), HLA-B∗57:01 and -A∗24:02 (residues 144, 151, 116, 113/114/116, 95/97), and HLA-A∗24:02 and -A∗25:02 (residues 90, 149, 152), and the replacement of KIR3DL1 contact residues on HLA-A∗24:02 and -B∗57:01 with alanine (residues 16, 17, 18, 72, 76, 79, 80, 83, 84, 89, 142, 145, 146, and 151) or glycine (149 and 150) (18) as well as our unpublished data examining residues 62 and 109, and comparing HLA-B∗27:04/05/06 recognition (42).

**Figure 7:**
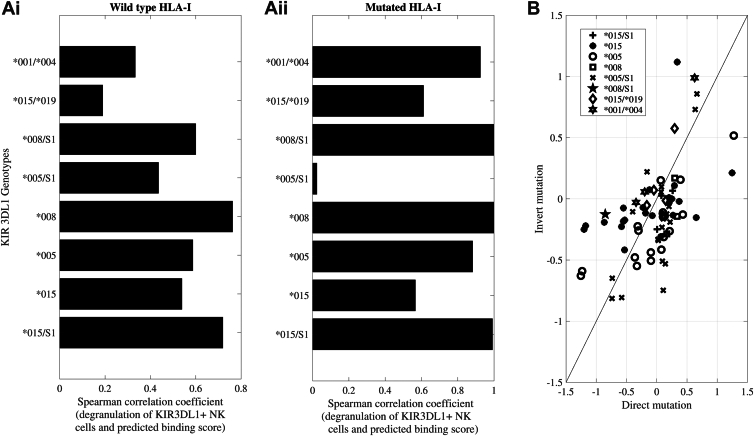
**Predicted binding correlates with KIR3DL1+ NK cell recognition of HLA-I-expressing targets.***A*, correlation between prediction based on binding data and observed degranulation of primary KIR3DL1+ NK cells towards target cells expressing (*i*) wild type HLA-I molecule*s* and (*ii*) HLA-I mutated at specific residues. For heterozygous KIR3DL1+ NK cells, the average predicted binding for the two expressed KIR3DL1 allotypes was computed. *B*, comparison of the effect of opposite mutations on degranulation. Each symbol represents KIR3DL1+ NK cells expressing a different allotype. Each point represents the change in binding affinity of a mutation (*e.g.* Q- > K in position 144) and minus the effect of the opposite mutation (K- > Q in position 144 on a different allele background). If the contribution of different amino acids was additive, all points would be expected to be on the diagonal.

An expected KIR3DL1/HLA-I binding affinity was computed for each HLA-I and mutant based on its amino acid sequence. Since individuals can express more than one KIR3DL1 allele, the expected binding was computed as the average binding for all the expressed KIR3DL1 alleles in the individual. To control for variability across HLA-I mutants and experiments, the observed degranulation of KIR3DL1^+^ NK cells was first normalized to their response towards the HLA-deficient 221 parental cell line in each assay (representative of the maximal degranulation). This value was correlated with the computed binding affinity for the wild type HLA-I molecules or their respective mutants (Fig. 7*A*). In both cases, a positive correlation was observed for all KIR3DL1 allotypes with an average of 0.4. Although the variable sample sizes here contributed to the differences in correlation, a higher accuracy was obtained for KIR3DL1 homozygotes.

The model used here presumes a linear contribution of each amino acid to the log of the binding strength. As such, mutation of a given amino acid would be expected to have an opposite log ratio change upon mutation in the reverse direction (for example, one could mutate HLA-A∗24:02 with a Q- > K mutation or HLA-B∗57:01 with a K- > Q mutation at position 144 – if each position was independent, the two were expected to have precisely opposite effects). To test how consistent the linearity assumption was, the log ratio of the degranulation of KIR3DL1^+^ NK cells toward a given HLA-I mutant (direct mutation) was correlated with the log ratio of the reciprocal mutation (invert mutation) (Fig. 7*B*). When assessed by KIR3DL1 allotype, the responses of KIR3DL1∗005^+^ NK cells to reciprocal HLA-I mutations showed higher correlation to each other than the response of KIR3DL1∗015^+^ NK cells to such mutations. Given that the binding affinity of KIR3DL1∗015 displayed greater variability across HLA-I than KIR3DL1∗005 (Fig. 2), the sensitivity of KIR3DL1∗015 to these reciprocal mutations is likely affected by its capacity to bind HLA-I of different allotypes. In contrast, the broader recognition of HLA-Bw4 allotypes by KIR3DL1∗005, as well as its greater peptide tolerance (15) may allow for more reflective impacts by individual HLA-I mutations. These results highlight both the strength of the general prediction model developed here and some of its limitations.

### Web tool

A web tool has been developed (https://kir-hla.math.biu.ac.il) that allows input of either an HLA-A, -B, or -C allele name or the amino acid sequence of an allotype (including novel or hypothetical alleles not defined in the nomenclature) and predicts their likely binding to KIR3DL1 allotypes. The output from 11 models is provided as described in the methods along with an indication of how this allele performs relative to the full list of HLA-A, -B, and -C alleles evaluated. The matrix of beta values for all 11 models is available for download as well as the amino-acid encoding for 15,474 HLA-A, -B, -C alleles (as of 2023–04–01) (Figs. S1–S4).

## Discussion

The human immune system relies on interactions between two highly variable families of genes: HLA, which presents protein fragments to immune cells, and KIR, which helps regulate how natural killer (NK) cells respond. These interactions are essential for recognizing infected or cancerous cells, and even small genetic differences can influence health outcomes. However, because there are thousands of variations in both HLA and KIR genes, it is not feasible to study all possible combinations in the laboratory. As a result, many clinical studies rely on simplified models that may overlook meaningful variation. Our study addresses this gap by using machine learning to predict how diverse KIR3DL1 receptors interact with different HLA class I allotypes, including those that have not been experimentally characterized.

While binding assays using KIR tetramers or Fc proteins with HLA-coated beads have greatly expanded the number of measurable KIR/HLA interactions, the full diversity of HLA and KIR across global populations is far too large to be captured through direct testing. In fact, even in well-studied populations, fewer than 30% of individuals carry KIR3DL1 and HLA-I allotypes for which direct binding measurements are available. To improve coverage, we characterized the binding of nine KIR3DL1 allotypes to 97 HLA class I molecules and trained a machine learning model to predict binding across all known HLA-I sequences.

**Figure 8:**
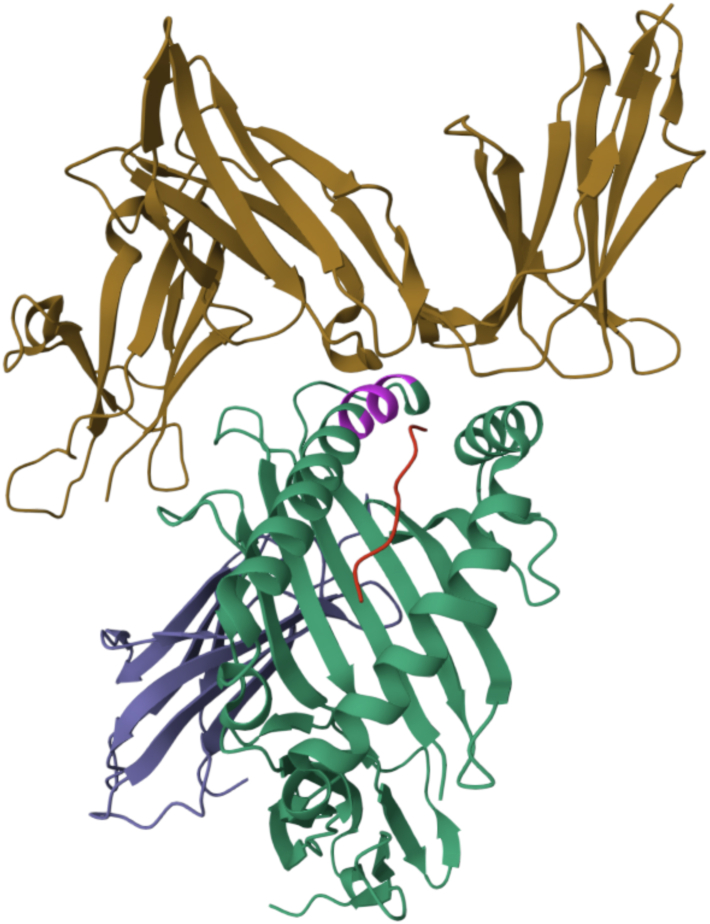
**Structure of 3DL1∗001 (*brown*) and HLA-A∗24:02 (*green*) with RYPLTFGW peptide(*red*) (PDB accession****7K80****).** The Bw4 region is highlighted in *pink*. The Bw4 region is directly interacting with the 3DL1. However, other positions, distant from Bw4, contact the 3DL1 molecule.

From this analysis, three key findings emerged. First, KIR3DL1 binding to HLA-I allotypes falls into three categories—high, low, and non-binding—rather than a simple binary classification. Second, although the Bw4 motif at positions 77 to 83 remains a strong predictor of binding, additional residues, particularly on the α1 helix, significantly influence interaction strength (Fig. 8). Third, while most KIR3DL1 allotypes have similar binding profiles, certain variants, such as KIR3DL1∗004 and ∗005 show distinct patterns. These allotypes recognize a broader range of HLA molecules and rely more heavily on non-Bw4 residues. In contrast, other allotypes such as ∗015 and ∗029 are fastidious in binding the classical Bw4 motif.

These predictions are consistent with prior studies showing KIR3DL1∗005^+^ NK cells to have broader HLA binding capacity and greater peptide tolerance (9, 15), and likely explains why the simple linear predictor obtained higher accuracy on other KIR3DL1 allotypes. The model’s feature coefficients at the Bw4 motif are consistent with detailed mutagenesis experiments (9, 15, 16, 41), while also capturing added predictive value from distal positions.

KIR3DL1 can also interact with the peptides bound by HLA class I molecules, which can further influence binding strength (14, 43). For instance, although HLA-B∗57:03 and HLA-B∗57:01, differ only at positions 114 and 116 in the floor of the peptide binding groove (17), they exhibit distinct effects on KIR3DL1 engagement due to differences in how the same peptide is presented. Developing models that can accommodate these complex peptide sequence/confirmation effects will be challenging. It is important to note that the HLA-I molecules used in our binding assays are expressed from recombinant cell lines and present diverse, endogenous peptide repertoires (44). The observed binding hierarchies in these assays correspond with functional NK cell responses, suggesting that the model captures general features of KIR recognition. Notably, peptides appear to exert greater influence in weak HLA binders, where optimal contact with the HLA scaffold is lacking (18) [Saunders, et *al in press*].

These findings have clear implications for research and clinical applications. KIR3DL1-HLA class I interactions influence immune responses in a range of settings, including hematopoietic cell transplantation (25, 26, 45, 46), viral infection (3, 29, 47, 48), cancer immunotherapy (49), and even neurological disease (50, 51). Yet clinical studies often reduce this complexity to the presence or absence of the Bw4 epitope or specific residues like I80. Our model provides a more detailed understanding. For example, although HLA-A∗25:01 carries the I80/R83 motif, it is a poor KIR3DL1 binder—a nuance captured by our model but overlooked in binary categorizations. This enhanced resolution could improve the interpretation of prior studies. In transplantation research, for example, KIR3DL1 expression levels and Bw4 subtypes have been linked to relapse risk in AML patients. A study by Boudreau *et al.* found that “weaker” KIR3DL1-HLA pairings—defined using expression levels and residue 80—were associated with reduced relapse rates (25). However, follow-up studies failed to replicate these findings consistently (52), likely due to genetic and treatment variability across cohorts. Our model allows reanalysis of such studies using more precise definitions of KIR-HLA interactions, potentially uncovering effects that were previously missed. It also highlights the need to consider HLA-A alleles with Bw4 motifs, which are often ignored but can still inhibit NK cell function.

While our predictor performs well, certain limitations remain. For example, the model correctly predicted HLA-A∗25:01 as a non-binder despite I80, but misclassified HLA-B∗13:01 as a binder (Fig. S1), although it failed to engage KIR3DL1 in both binding and functional assays (18). Future improvements will need to account for structural flexibility and allotype-specific interaction mechanisms, as shown in recent studies of KIR2DL interactions with peptide-loaded HLA-C allotypes (37). These findings challenge the assumption that a given residue has a uniform role across all interactions.

Nonetheless, this model provides a powerful tool for predicting KIR3DL1 binding strength across the full range of HLA class I diversity. By offering allele-level resolution—including for under-characterized or novel variants—it enhances the utility of KIR/HLA genotyping in both research and clinical settings. As more experimental data become available, the model can be further refined and expanded. In the meantime, it supports more accurate analyses in transplantation studies, disease association research, and the design of NK cell-based immunotherapies.

## Experimental procedures

### KIR3DL1/HLA-I binding affinity measurements

HLA-I recognition by KIR3DL1 allotypes was assessed through binding of KIR3DL1 tetramers to beads coated with a panel of 100 different HLA-A, -B, and -C molecules (LABScreen HLA Class I Single Antigen; One Lambda). These experiments have been described in detail previously (9). KIR binding affinity values were determined over a series of 3 runs per KIR3DL1 allotype. The results were averaged and normalized against the maximal response to produce a matrix of raw binding values for each HLA-I and KIR3DL1 combination, distributed between 0 and 1.

### 2D-clustering

The log of the raw matrix above was clustered using hierarchical clustering with average link clustering on both the KIR3DL1 and the HLA-I allotypes using a Euclidean distance between samples. The results presented are ordered following the clustering in both directions. For HLA-I clustering, each HLA-I was represented by their nine-dimensional normalized binding vector of affinities to the KIR3DL1 allotypes. The opposite was performed for the KIR3DL1 allotype representations.

### Logo plots

Logo plots were computed using the Two Sample Logo web tool (53). Sequences were grouped into three clusters of HLA-I allotypes based on the hierarchical clustering: high, low and non-binding. All sequences were then aligned, with the high and low binding levels compared with the non-binder clusters, and the low- and high-binding groups compared one to each other using a binomial test. The presented residues represent the amino acids with a significant difference at a 0.05 level with a Bonferroni correction (54).

### Machine learning and regression

The amino acid sequences of the different HLA-I allotypes were converted into amino acid-position pairs (for example R6 represents an arginine at position 6). Positions for which there was no variability in all HLA-I allotypes studied were removed from the samples. Each HLA-I allotype was then represented as a binary vector with 1 in the appropriate position if the HLA had the corresponding amino acid. For example, an HLA that starts with RA would have values of 1 in positions R1 and A2, and 0 in all other amino acid possibilities for position 1 and 2 (*e.g.*, S1 and L2). This is a one-hot representation with non-polymorphic encodings removed (further denoted as one-hot vectors). Linear or kernel-based predictions were then applied on the log of the binding affinity. Zero values were replaced by a minimal value (1% of the minimal positive value to avoid a log of zero values).

The prediction was performed using four different regions and/or residues of the HLA-I: 1) all positions in the second and third exons of the HLA-I allele; 2) only the six residues associated with the Bw4 motif (defined as [76:77 80:83]); 3) no loops (where the loops were defined as [16:19,39:44,49:56,86:90,106:107,128:131,137:140,151:152,176:179,180:183]); and 4) residues encoding only the alpha helices (defined as [57:85,141:175]) or as the exon 2 and 3, without loops. When the regression was performed only on the Bw4 positions, the one-hot vectors themselves were used. In all cases, first either a Multiple Correspondence Analysis (MCA) (55) or a Principal Component Analysis (PCA) were performed over all the one-hot vectors. Then machine learning was performed on the projection over the first K MCA vectors (*i.e.* projection to K dimensions). In the current application, K was set to be seven, based on the decrease in the contribution to variance beyond the seventh Eigenvector. The results presented for the KIR3DL1 binding prediction are an average over five cross validations. In all learning tasks, a 5-fold cross validation was performed where the percentage of training and test samples was 80% and 20% respectively, unless explicitly stated otherwise. For each cross-validation, each HLA-I allele was either always in the test or always in the training set for all KIR3DL1 allotypes. The following classifiers were used.

#### Support Vector Classification (SVM)

The implementation of this method was based on *libsvm* (56). The penalty parameter of the error term (the “Box Constraint”) was set to 0.01. The kernels tested were linear and polynomial and a “balanced” class weight was used, implying that the box constraint is normalized to be inversely proportional to the class size.

#### Multi Label Vector Optimization

Formally assumes samples have binary and continuous labels (not necessarily both labels for all samples). We assume that continuous observations are monotonically related to the binary classifications. Each sampled point can have either one of the two label types or both. When both are used the resulting kernel machines based optimization problem has been denoted the MLVO (41).

More complex methods, such as Random Forest, XGBoost, and Neural networks, had lower performance scores. The precisions of the classifiers were computed using the Area Under the sensitivity-specificity Curve (AUC).

### Phylogeny

For the comparison between different KIR3DL1 allotypes, the Hamming Distance (57) between their sequences was computed using the amino acid sequence over the entire KIR3DL1 coding sequence, following gene alignment between all the studied KIR3DL1.

### Web tool

A web tool (https://kir-hla.math.biu.ac.il/Home) was developed using the python flask framework that implements the predictions from the model based on the amino-acid sequence of the input HLA-I allele. The output is based directly on the matrix of beta values from 11 models: overall binding, high vs low binding, and KIR3DL1 allotype specific models for the 9 training allotypes: KIR3DL1∗001, ∗002, ∗004, ∗005, ∗008, ∗009, ∗015, ∗020, ∗029. Figures S1, S2 and S4 shows the response of the tool to an input for specific allotypes. Figure S3 shows the response of the tool to an input of an HLA Class I amino acid sequence. The web tool ignores amino acids that are missing (‘∗’).

### Statistical analysis

A two-sided two population *t* test was used to compare the log binding scores between the average of the T80/G83 group (minimal binding) and each other 80/83 group, with no multiple measurement corrections (Fig. 2, black stars). A one-way ANOVA between all groups was always significant at the *p* = 0.001 level. A *post hoc* Tukey test on each group vs each other group was further performed (Fig. 2, blue stars), All *p* values are reported in the Table S1. For comparisons between the AUC of modeled HLA regions (Fig. 5*B*, *i* and *ii*) a two-way repeated measurement ANOVA with the input type was performed and the loci included as independent variables. For the comparison between KIR3DL1 allotypes (Fig. 5*C*), a one-way ANOVA with the 3DL1 allele as an explaining variable was carried out. For the correlation between the reported KIR3DL1^+^ NK cell degranulation and the predicted score (Fig. 7, *A* and *B*), the expected binding score to each HLA and the log normalized degranulation level were computed for each set of experiments with the same surface expressed KIR3DL1 allotype, where each degranulation level was divided by its baseline, and computed for each such sets the Spearman Correlation Coefficient between the predicted score and the normalized degradation level. For the mutation experiments (Fig. 7*C*), the log ratio between the degranulation level before and after the mutation was calculated, and in parallel the expected score difference, and the correlation between the score difference and the log ratios computed.

## Data availability

This article contains supporting information. The datasets analyzed for this study can be found in Table S2.

## Supporting information

This article contains supporting information.

## Conflict of interest

The authors declare that they have no conflicts of interest with the contents of this article.
