# Supplementary material for: Prediction of KIR3DL1 and human leukocyte antigen binding
Source: J Biol Chem. 2025 Jul 1;301(8):110437. doi: 10.1016/j.jbc.2025.110437 (PMC12309609; doi:10.1016/j.jbc.2025.110437)
Supplement: Supplemental Figure and Table Legends [file mmc1.docx]

**Supplemental Figure Captions**

**Supplemental Figure 1: *Binding prediction for allele HLA-A*25:01.*Upper panel:** Red bar indicates the binding score; green bar shows the log-transformed score comparing high vs. low binders; blue bars represent individual binding scores for each KIR3DL1 allotype.
**Lower panel:** Comparison of binding score fractions under the same conditions. Solid bars represent the fraction of alleles with a lower binding score than HLA-A*25:01. The empty bar indicates the fraction of HLA alleles classified as high binders for a given KIR allotype. A solid bar taller than the corresponding empty bar suggests that the current allele is a high binder.

**Supplemental Figure 2: *Binding prediction for allele HLA-B*13:01.*Upper panel:** Red bar indicates the binding score; green bar shows the log-transformed score comparing high vs. low binders; blue bars represent individual binding scores for each KIR3DL1 allotype.
**Lower panel:** Comparison of binding score fractions under the same conditions. Solid bars represent the fraction of alleles with a lower binding score than HLA-B*13:01. The empty bar indicates the fraction of HLA alleles classified as high binders for a given KIR allotype. A solid bar taller than the corresponding empty bar suggests that the current allele is a high binder.

**Supplemental Figure 3: *Binding prediction for allele HLA-C*01:02.*Upper panel:** Red bar indicates the binding score; green bar shows the log-transformed score comparing high vs. low binders; blue bars represent individual binding scores for each KIR3DL1 allotype.
**Lower panel:** Comparison of binding score fractions under the same conditions. Solid bars represent the fraction of alleles with a lower binding score than HLA-C*01:02. The empty bar indicates the fraction of HLA alleles classified as high binders for a given KIR allotype. A solid bar taller than the corresponding empty bar suggests that the current allele is a high binder.

**Supplemental Figure 4: *Binding prediction for allele HLA-A*24:02.*Upper panel:** Red bar indicates the binding score; green bar shows the log-transformed score comparing high vs. low binders; blue bars represent individual binding scores for each KIR3DL1 allotype.
**Lower panel:** Comparison of binding score fractions under the same conditions. Solid bars represent the fraction of alleles with a lower binding score than HLA-A*24:02. The empty bar indicates the fraction of HLA alleles classified as high binders for a given KIR allotype. A solid bar taller than the corresponding empty bar suggests that the current allele is a high binder.

**Supplemental Figure 5: *Comparison of AUC across models and feature sets.*** Each subplot represents a different model. The left column includes models using only the HLA-A and -B loci, while the right column includes models using HLA-A, -B, and -C loci. Each row corresponds to a different positional feature set: the top row includes all positions, the second row includes only Bw4-associated positions, the third row excludes loop regions, and the bottom row includes only alpha helices. Within each subplot, we compare two dimensionality reduction methods (PCA and MCA) and two regression algorithms (SVM and MLVO). Overall, PCA tends to outperform MCA, and MLVO generally achieves higher average AUC values, though with greater variance.

**Supplemental Table Captions**

**Supplemental Table 1: *Additional statistics for comparison between the AUC of modeled HLA regions.*** All *p* values are reported for comparisons between the AUC of modeled HLA. A two-way repeated measures ANOVA was performed with input type as the repeated factor, and loci included as independent variables.

**Supplemental Table 2: *Raw binding affinity measurements.*** The normalized MFI values for the KIR3DL1/HLA-I binding assay are provided for three runs of each of nine KIR3DL1 alleles. Mean values were computed and normalized against the maximal response. Each row represents alleles in the panel with non-zero MFI in at least one of the three runs.
