# Supplementary figures and images for: Prediction of KIR3DL1 and human leukocyte antigen binding
Source: J Biol Chem. 2025 Jul 1;301(8):110437. doi: 10.1016/j.jbc.2025.110437 (PMC12309609; doi:10.1016/j.jbc.2025.110437)

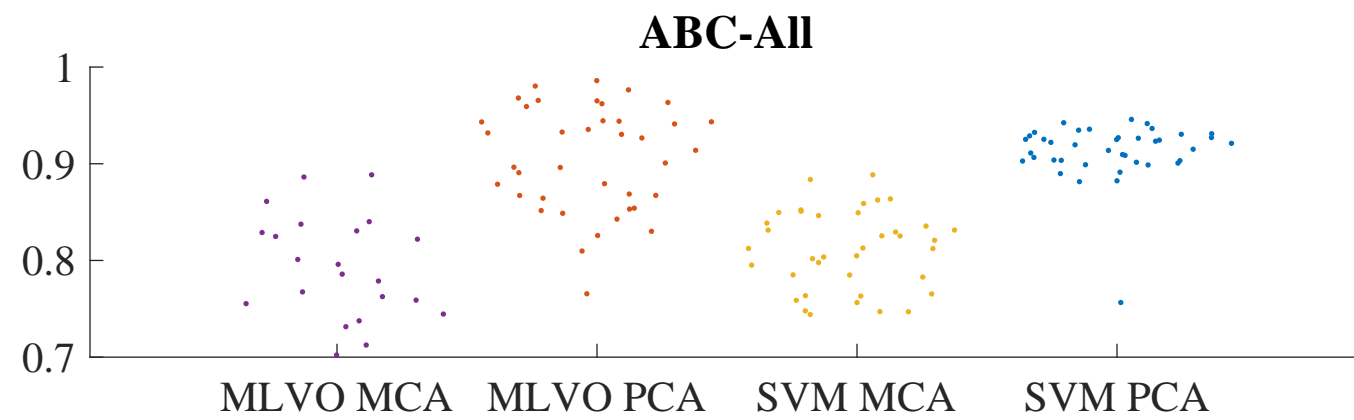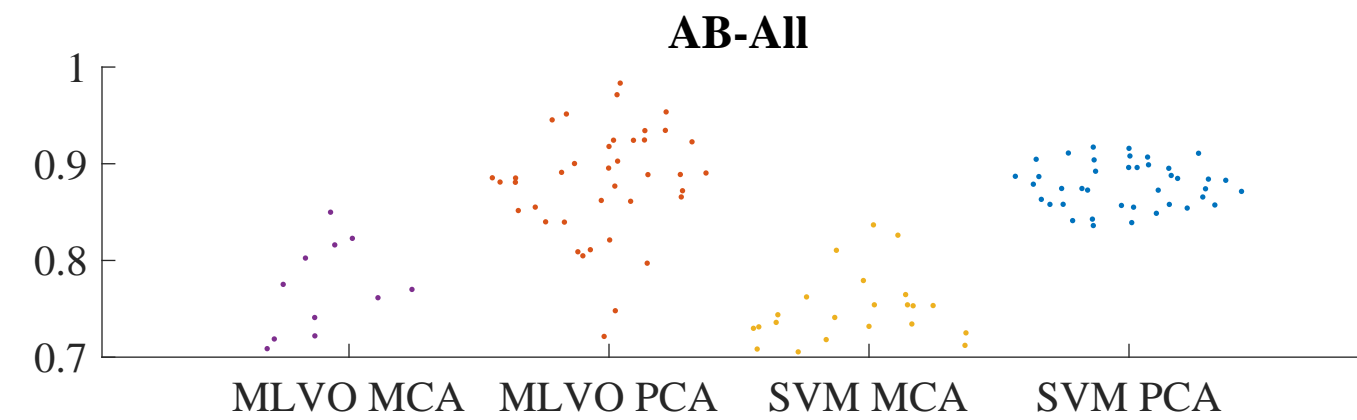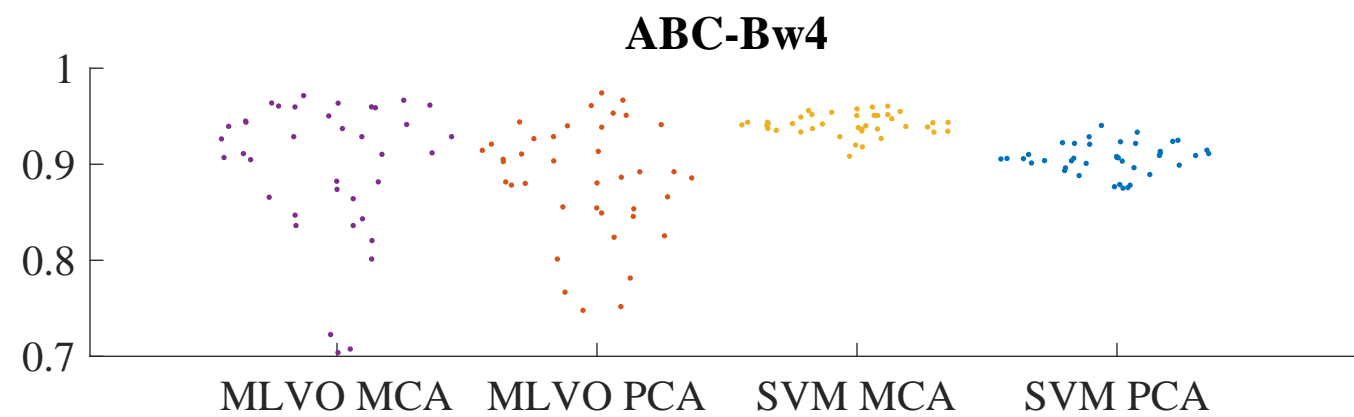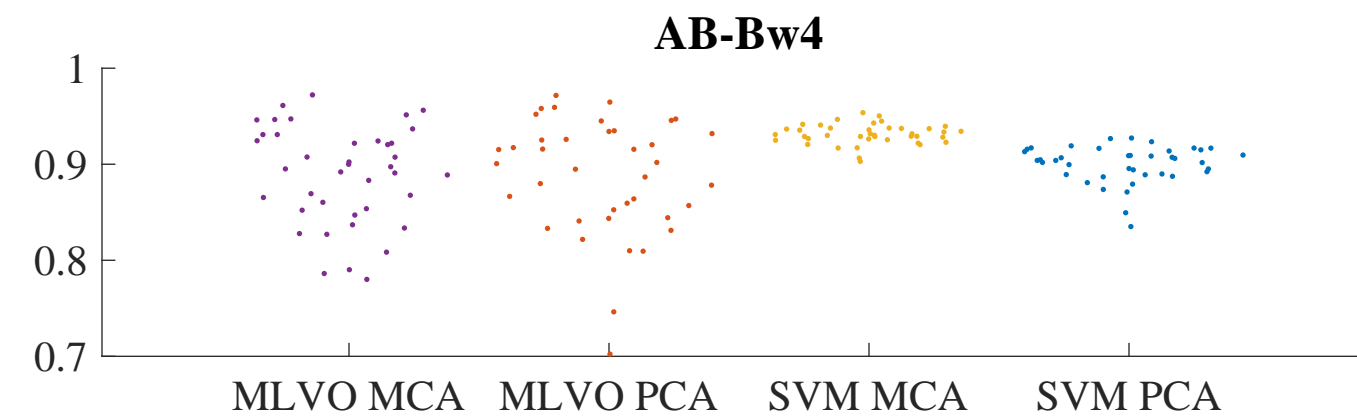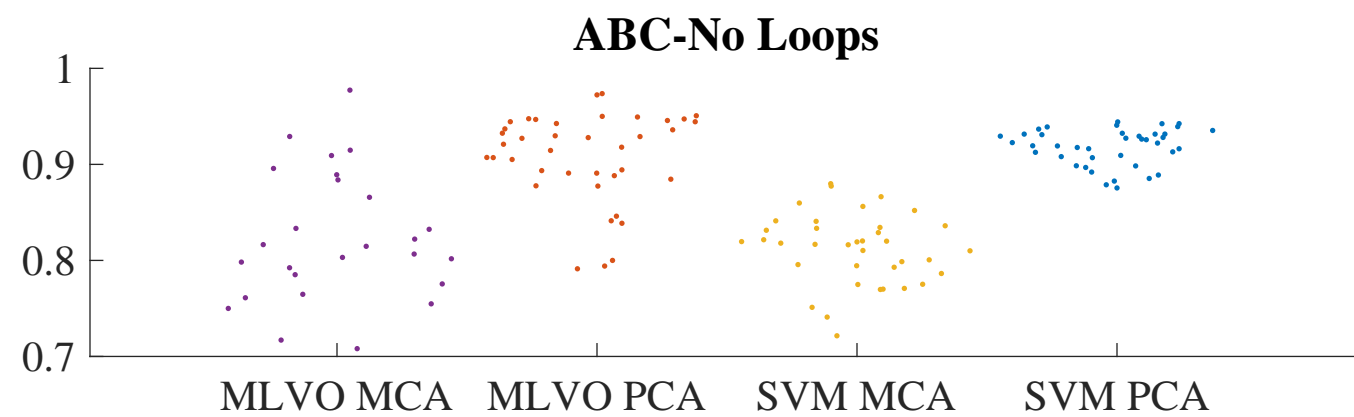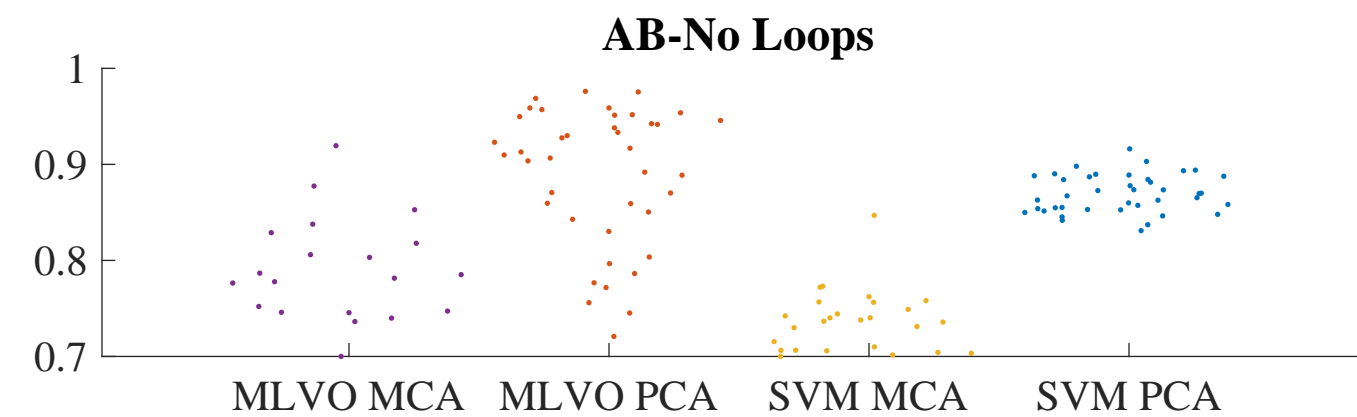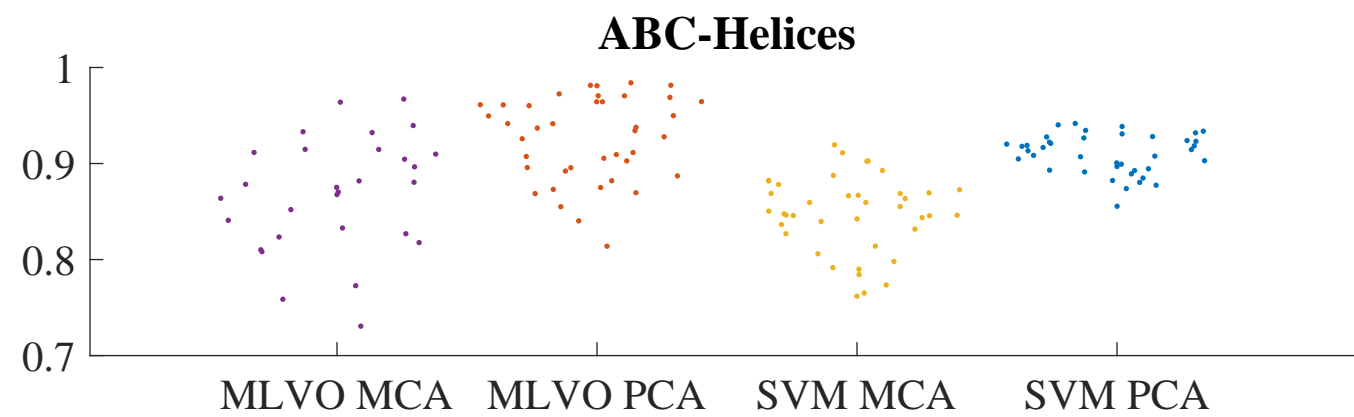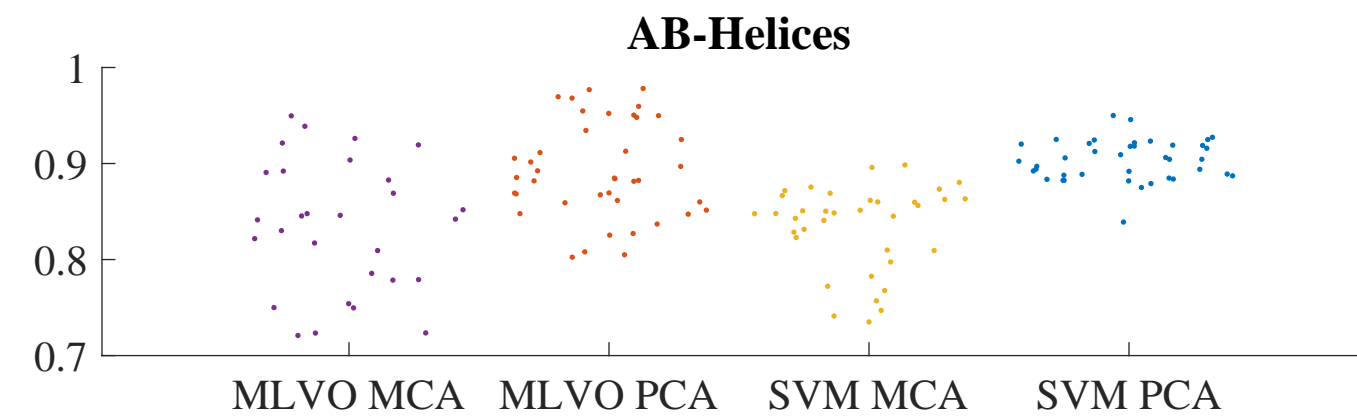

Supplement: Figure S5 [file mmc6.pdf]
